# University of Louisville

**Published:** 1854-06

**Authors:** 


### UNIVERSITY OF LOUISVILLE.

IT was announced by us, some months ago, that PROF. SILLIMAN had resigned the chair of *Medical Chemistry and Toxicology* in the University of Louisville. We have the pleasure to inform our readers that the vacancy thus created has been filled by the election of J. LAWRENCE SMITH, M. D., formerly of Charleston, South Carolina, and lately Professor of Chemistry in the University of Virginia. PROF. SMITH is extensively known as a successful cultivator of chemical science. His researches, both in chemistry and mineralogy, have been numerous and important. Although now devoted to chemistry as a pursuit, he was regularly educated for the profession of medicine, his acquaintance with which will enable him to point out to his students the many intimate relations of chemistry to medical science. It is his intention to settle permanently in this city where, before he was elected to this chair in the University, he had erected a laboratory for the purpose of pursuing his scientific investigations. Prof. Smith, we have no doubt, will prove an acceptable teacher, while as a practical, working chemist, and gentleman of varied, profound science, he is an acquisition to the city.

From Prof. Flint's letter, which forms an interesting part of our present number, it will be seen that this gentleman is pursuing his medical observations with great industry and zeal in the hospitals of Paris. He writes to his colleagues in the University, that he will have it in his power to make valuable additions to the Anatomical Museum from the rich collections of the French capital. He will return to Louisville about the beginning of October.

